# Digital clinical trials: creating a vision for the future

**DOI:** 10.1038/s41746-019-0203-0

**Published:** 2019-12-12

**Authors:** Steven R. Steinhubl, Dana L. Wolff-Hughes, Wendy Nilsen, Erin Iturriaga, Robert M. Califf

**Affiliations:** 1 Scripps Research Translational Institute & Wave Research Center, LaJolla, CA USA; 2 National Institutes of Health, Office of Behavioral and Social Sciences Research, Bethesda, MD USA; 3 National Science Foundation, Computer and Information Science and Engineering, Alexandria, VA USA; 4 National Institutes of Health, National Heart, Lung, and Blood Institute, Bethesda, MD USA; 5 Verily Life Sciences, Alphabet Inc, South San Francisco, California, USA and Duke Forge, Duke University School of Medicine, Durham, NC USA

**Keywords:** Clinical trials

Digital technologies have transformed almost every aspect of our lives over the past decade including the way we communicate, shop, and read. Digital health technologies, despite their reputation for over-promising and under-delivering, can potentially offer the solutions needed to transform clinical trials, if backed by sufficient investment and regulatory support. However, this cannot be accomplished by replicating the current research processes and just transforming them from paper to digital form. Rather, a complete re-thinking and re-engineering of the clinical trial experience around the participant rather than the research site is needed. While some trials could be entirely digital in a virtual environment, many will need a hybrid of virtual and clinical site-based activities.

Clinical trials are the central mechanism for unbiased assessment of proposed advances in health, healthcare, and evaluation of comparative options for approaches to prevention, diagnosis, and treatment. For trials to inform clinical decision making and to be of greatest value, the setting should be consistent with clinical practice and participants should be representative of the individuals who will use the new therapies or delivery approaches. Unfortunately, the existing clinical research infrastructure is changing only gradually, and that often leads to clinical trials being both logistically challenging and excessively expensive.

There is ample evidence of the need for a better solution to the current clinical trial system. For example, while the public broadly supports and values clinical research, <10% of eligible individuals are asked to participate.^1,2^ The additional time and travel commitments too often required for clinical trial participation can lead to many interested people declining the opportunity. Clinicians are likewise dispirited by the numerous repetitive practices required in the current clinical trials enterprise, many unnecessary if available digital data sources were to be fully used, leading them to avoid asking patients to participate. This limited enrollment can lead to health decisions that are too often informed by results obtained in an artificially homogeneous population,^3^ or made in a vacuum of high quality evidence.^4^ Related to the lack of diverse participants is the agonizingly slow enrollment that most clinical trials experience, typically requiring nearly double the time of what was planned, with half of clinical sites enrolling none or very few participants.^5^ This, plus other factors, contribute to what is often the greatest barrier to clinical trials, the costs, which can routinely range into hundreds of millions of US dollars.^6,7^

Data from electronic health records and claims data, which is already collected as part of routine care combined with real-world signals from mobile phones, wearables, implanted and in-home sensor technology will enable remote, continuous monitoring of participants as the data quality and types improves, thereby eliminating most travel requirements to a clinical site. These changes will also allow for more frequent and real-time follow-up of participants, overcoming the limitation of in-clinic exams as the primary means of objective follow-up. In addition, innovative sensors can provide novel data to more precisely refine phenotypes, such as continuous glucose monitoring.^8^ Another unique capability digital technology brings to trials is near ubiquitous, 24/7 connectivity fostered by smart phones and especially cell phones (owned by 96 and 81% of adults living in the United States, respectively), which enables two-way, real-time communications from most physical locations.^9^ Besides also helping to minimize geographic obstacles to participation, this level of connectivity allows for the possibility of individual findings and overall results to be returned to participants throughout the duration of the study, fostering a true partnership in research. A transformational aspect of digital trials is how they can truly involve participants as partners and even enable patients to design and lead clinical trials.^10^

To manage and make sense of the vast amounts of data capable of collection through these novel techniques will require the broad use of another digital technology: artificial intelligence (AI). Combined with traditional biostatistical methods, AI will be useful to tackle daunting problems of missing data and artifact that lead to questions about interpretation of digital data. The user interface and communication around technology implementation will likely require a specific, different role for research sites in assuring fidelity of “real world” measurements and increasing use of in-home visits (virtual and in-person) to ensure that high quality data are being collected.

In order to chart a path forward for greater implementation of digital clinical trials, a workshop co-sponsored by the National Institutes of Health (NIH) and the National Science Foundation (NSF) was recently convened to discuss the challenges and necessary next steps to move the field forward. (See https://www.nhlbi.nih.gov/events/2019/digital-clinical-trials-workshop-creating-vision-future).

There are substantial, although undoubtedly addressable challenges. The characteristics of digital technologies that make them attractive for use in health research- their pervasiveness and depth of easily captured information- also make the data they generate valuable to commercial entities as well as to malign actors. Frequent news of major data breaches, or stories of hidden app trackers sharing personal data without the user’s knowledge reinforce security and privacy concerns of researchers and participants alike.

While trust is often inherent between patients and clinicians and supported by legal guidance such as the Health Insurance Portability and Accountability Act, the lines are blurry or nonexistent for commercial entities with health-related technologies or those on the fringes of health like social media. This lack of oversight enabled the majority of app developers to broadly share data without the user’s explicit knowledge or consent.^11^ In Europe the General Data Protection Regulation was recently enacted to protect broadly defined data, including health data, and to mandate control and choice in accepting or declining terms of service (http://www.privacy-regulation.eu/en/index.htm). Because the preservation of confidentiality is mandatory for maintaining the trusting relationship needed in medical research, at a minimum a digital research infrastructure must achieve the confidence level of other sectors dealing with private and highly sensitive information digitally, such as finance and banking. Increased future use of novel data structures that provide a verifiable and tamper-proof history of all transactions can offer greater assurance of data security.^12^

The ethical basis of human experimentation has been driven by awareness of exploitation of vulnerable individuals and populations. This exploitation has led to a complex framework built on Institutional Review Board oversight at the level of the research site, which is defined by attested accountability of a site principal investigator and a sponsor for appropriate conduct within a reviewed protocol. The U.S. Food and Drug Administration regulations reinforce this approach with significant penalties for failing to follow the federal regulations. Continued partnership with regulators will be needed as increasing real-world experience surfaces unanticipated complexities in order to maintain the balance in favor of benefit and reduce the risk of exploitation in the digital sphere.

Beyond concerns with security, privacy, and data quality, a significant barrier to implementation of digital clinical trials is the process of participant recruitment, enrollment, and follow-up that have become revenue generators for research centers. To foster change, many existing research organizations, both commercial and academic, will face the same difficulties that a number of corporations have been forced to address over the last decade dealing with their own digital disruption (Table 1). However, unlike personal photography, travel, retail sales, and many more industries driven by consumer preference, ongoing funding of clinical research depends almost solely on the decision of trial funders, whether grant reviewers or medical industry leaders, who historically tend to support the status quo rather than drive innovation.^13^

**Table 1:** Examples of organizations and industries that have undergone digital disruption or have successfully transformed themselves by implementing digital solutions.

Industry or organization	Core business	Transformational change	Digital disruption enhance existing income model?	Successful internal transformation?	Examples of digital disrupters
Photography supplies	Photographic film & paper	Digital photography	No	×	Sony
Book stores	In person selection & selling of books	Online book search & sales	No	×	Amazon
Financial institutions	In person money lending	ATMs and online banking	Yes	✓	–
Video & DVD rental	In person selection & rental	Digital browsing and streaming	No	×	Netflix
Traditional clinical research organizations	In-person recruitment & management of study participants	Participant-centric digitally-enabled clinical trials	No	?	Apple ResearchKit

The traditional clinical research enterprise should take action to benefit from digital disruption, rather than attempting to maintain the status quo, which has made numerous traditional entities obsolete

So, what is needed to overcome existing challenges and drive innovation using digital technologies? As noted earlier, establishing standards and protocols supporting transparency and privacy of participant’s data is critical. Next, incentives for clinical trials that necessitate the development of innovative solutions to existing problems in health research are needed. For example, providing funding opportunities for nationwide trials targeted to historically hard-to reach populations, such as those living in a rural setting, or supporting programs designed to refine existing knowledge of inexact phenotypes such as hypertension, diabetes, or anxiety disorders based on the use of validated, personal sensors used continuously over prolonged periods of time in a real-world setting. Importantly, ongoing learning and near real-time adaptability, a major advantage of clinical trials using digital technology, will need to be an anticipated and supported component of any funded program as their novelty will come with many unanticipated lessons.

Improving health for the public is central to the mission of both the NIH and NSF with multiple efforts highlighting a digital clinical trial focus, including NIH’s commitment to the use of FHIR standards for federally-funded clinical trials (https://grants.nih.gov/grants/guide/notice-files/NOT-OD-19-122.html). Other examples include the NIH-funded Eureka Platform to support the development of valid and reliable mobile technologies and the Intensive Longitudinal Health Behavior Network to develop models of human behavior that can then be used for health monitoring. Further, the joint Smart and Connected Health program (NSF-18-541) supports the advances in technology needed to drive digital clinical trial development. Despite these efforts, more needs to be done. Affordable, rapid, pragmatic, and participant centric clinical trials are needed to accelerate that advancement.^14^ Digital technologies, although in the early days of implementation in health research, offer unique tools critical to this transformation.

Both patients and clinicians are insistent that we need better and faster evidence to inform decisions. While market forces drove digital disruption in many other industries, the clinical research community, funding agencies, and regulators will need to work together, to encourage methodological innovation and develop a digital clinical trial enterprise.

Disclaimer: Any opinions, findings, and conclusions or recommendations expressed in this paper are those of the authors and do not necessarily reflect the views of the NHLBI or the NIH.
